# Blood transfusion services in Bangladesh

**DOI:** 10.4103/0973-6247.53880

**Published:** 2009-07

**Authors:** Muhammad Badrul Islam

**Affiliations:** *Department of Blood Transfusion, NICVD, Dhaka Sher–E–Bangla Nagar, Dhaka -1207, Bangladesh*

**Figure 1:**
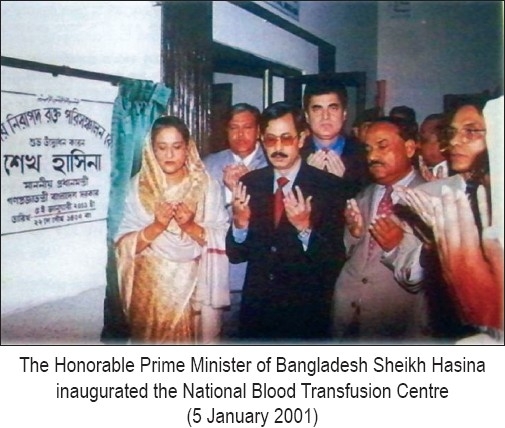
The Honorable Prime Minister of Bangladesh Sheikh Hasina inaugurated the National Blood Transfusion Centre (5 January 2001)

Bangladesh is one of the most densely populated countries in the world having a population of about 140 million. The Constitution of Bangladesh provides healthcare rights to every citizen. The government is determined to extend basic healthcare delivery to all citizens. According to the United Nations mandate the Government of Bangladesh (GoB) is committed to attain health-related Millennium Development Goals (MDGs) on child mortality, maternal mortality and Human Immunodefficiency Virus (HIV)/AIDS, TB, malaria and other disease prevention by the year 2015.[1] According to the statistics of the year 2007, in Bangladesh the density of population per square kilometer is 941.[1]

The land area of Bangladesh is 147,570 sq. km. Bangladesh is situated in the eastern part of the South Asian subcontinent. The country is bordered by India on the east, west and north and by the Bay of Bengal and a small border strip with Myanmar on the south.

Blood Transfusion Service (BTS) is an integral and indispensable part of the healthcare system now. The priority objective of BTS is to ensure safety, adequacy, accessibility and efficiency of blood supply at all levels.[2] BTS in Bangladesh started in 1950 at the Dhaka Medical College Hospital. In the year 1968, three more blood transfusion centers were opened at Midford Hospital, Dhaka; Chittagong Medical College Hospital and Rajshahi Medical College Hospital. Later, blood transfusion Departments became operational in different hospitals. In the year 1992 the government constituted a committee called “Blood Transfusion Committee” to offer advice on the introduction of donor selection criteria, matters relating to blood safety and introduction of various tests and other technical issues. In 1976 the “Bangladesh Council of Blood Transfusion Service” was established to supervise and monitor improvement of BTS in the country. A postgraduate diploma course in Transfusion Medicine was started in Bangabandhu Sheikh Mujib Medical University (BSMMU), Dhaka in the year 1972 for manpower development

The importance of ensuring blood safety as well as the adequacy of the national blood supply is highlighted due to the emergence of HIV in the 1980s. The global burden of diseases due to unsafe blood transfusion can be eliminated or substantially reduced by adopting an integrated strategy for blood safety. In the year 2000 the Government of the Peoples Republic of Bangladesh with assistance from UNDP, has launched a blood screening program all over the country through 97 blood transfusion centers from the districts towards the national level hospitals. The general objective of the program is to provide safe blood and blood products countrywide. There were specific objectives like1. Capacity-building of institution and service providers; 2. Ensuring institutional facilities of screenings and enhancing the spirit of voluntary blood donation. The following screening tests were performed in the blood transfusion centers:- HBsAg, VDRL, Malarial Parasite, Anti-HCV, Anti-HIV.

During that time we were dependent on professional donors rather than voluntary donors. In the year 2000, 47% blood was collected form professional blood donors, 27% was collected from family replacement donors and 26% blood was collected from voluntary non-remunerated blood donors.[3]

As the number of voluntary donors was less, there were a large number of professional donors. The prevalence of transfusion-transmitted infections (TTI) was high in professional donors. A study conducted by Prof. Md. Nazrul Islam and Prof. M. Hussain (unpublished data) showed TTI as mentioned in Table 1.

**Table 1:** Prevalence of infectious markers in blood donors

Donor Types	HBsAg	VDRL	Anti-HCV	Anti-HIV
Professional Blood Donor	29%	22%	6.2%	Nil
Voluntary Blood Donor	4%	0.9%	Nil	Nil
General Population	9%	1%	3.4%	Nil

## Prevalence of TTI in different populations in the year 2000[3]

The availability of various donor populations is gradually changing. Total number of blood units screened in 97 centers from January 2001 to May 2003 were 3,23,642. Out of them, 18.01% were paid donors, 26.01% were voluntary donors and 55.98% were family replacement donors. In a pilot study, 6143 units of TTI-positive blood were evacuated and HBsAg-positive units were 4,467 (72.72%) in number; Hepatitis C was positive in 466 units (7.59%); syphilis was positive in1,120 (18.23%); malaria was positive in 83 (1.35%) and Hive sero-positivity for HIV was observed in seven blood donors (0.11%).[2]

Through safe blood program, the government has successfully changed the previous picture. The number of paid donors was reduced from 70% to 10%. From 2001 to 2006, total 6,66,369 units of blood were screened. During this period 34 units were found seropositive for HIV infection among blood donors. All the seropositive units were tested again in the Reference Laboratory and Virology Department of BSMMU and finally only two were only two were positive for HIV infection.[4] Screening report of a blood donor of the year 2007 and 2008 has been given in Tables 2 and 3.

**Table 2:** Screening Report of the Year 2007

Donor Group	Total	(%)	HIV +ve		HBV +ve		HCV +ve		RPR +ve		MP +ve	
	Screening		No	%	No	%	No	%	No	%	No	%
Voluntary non-remunerated blood	94,100	29.04	9	0.0028	822	0.2537	60	0.0185	50	0.0154	3	0.0009
Relative blood donor	196,057	60.51	16	0.0049	1,887	0.5824	148	0.0457	126	0.0389	7	0.0022
Others	33,848	10.45	2	0.0006	55	0.0170	43	0.0133	39	0.0120	1,003	0.3096
Total	324,005		27		2,764		251		215		1,013	

**Table 3:** Screening Report of the Year 2008

Donor Group	Total	(%)	HIV +ve		HBV +ve		HCV +ve		RPR +ve		MP +ve	
	Screening		No	%	No	%	No	%	No	%	No	%
Voluntary non-remunerated blood	96,572	27.50	5	0.0014	1,045	0.2976	112	0.0319	65	0.0185	2	0.0006
Relative blood donor	250,117	71.22	7	0.0020	1,872	0.5330	170	0.0484	59	0.0168	1	0.0003
Others	4,499	1.28	0	0.0000	20	0.0057	16	0.0046	18	0.0051	0	0.0000
Total	351,188		12		2,937		298		142		1,013	

Source:- Program office Safe Blood Transfusion Program DMCH, Dhaka

## National Blood Transfusion Services

### Department of Transfusion Medicine (Blood Transfusion)

World Blood donor day, i.e. 14 June is always observed in Bangladesh. The world blood donor day is a celebration of voluntary, unpaid blood donors who enthusiastically give off themselves, to improve and save lives. By celebrating the day, the Bangladesh Government recognizes the special lifesaving role of voluntary non-remunerated blood donors and expresses its gratitude to all selfless donors.

The situation of BTS is improving day by day in comparison to the past. The growing problem of TTI has been acknowledged in Bangladesh like other countries of the world. As an integral part of the healthcare system, the objective of BTS of Bangladesh is to ensure safety, adequacy, accessibility and efficiency of blood supply at all levels. To reach this goal, the Bangladesh Government has passed “Safe Blood Transfusion Law 2002” in the Parliament and “Safe Blood Transfusion Rules 2008”, also published recently in June’08. This law is a regulatory law for setting up blood transfusion centres, management, blood collection, blood storage, blood testing and transfusion to prevent unauthorized practices of human blood transfusion. Establishment of private blood banks, operation, licensing system, inspection committee and punishment for violation of rules etc. is clearly stated in the law.[5]

Like other developing countries, blood banking in Bangladesh does not get enough attention for development from authorities. Many blood recipients remained at risk of TTI transmission as a result of poor blood donor recruitment and the use of low-quality testing in TTI screening. Considering the disease scenario and the importance of blood safety one project (TAPP) was approved in 1998 by the Ministry of Health and Family Welfare in the name of “Implementation of Safe Blood Transfusion”.[6] Later it was included as a part of “Safe Blood Transfusion Program”. The Honorable Prime Minister of Bangladesh Sheikh Hasina inaugurated the program office and this reflects the firm determination of the government to ensure safe blood for all and to develop the discipline. As per National regulation, there is a high-powered committee named “National Safe Blood Transfusion Council”. The President of the committee is the Health Minister and the Vice-President is the Secretary of Health. The Director General of Health Services is the Secretary General of the Committee. The other members of the committee are from different spectrums of the society including voluntary donor organizations. This council is regulating the blood transfusion services and making different rules for safe blood transfusion. In the new gazette, two new committees have been fromed. One of them is the National Blood Transfusion Expert committee which is bing headed by he Director General of the Health Services and members are drawn from the Transfusion Medicine departments. This Committee will implement the decision of the council. The other committee is the “Blood Transfusion Management Committee’ and this committee wil be constituted in all government hospital. The Chairperson of the committee is the Director of the respective hospital and the Vice-Chairperson is the Deputy Director, two imminent social workers and departmental heads/professors of different disciplines will be the members of this committee. The Head of the department of Transfusion Medicine will be the Member Secretary of the committee. All such committees has given financial autonomy to purchase carry out emergency purchase for blood banks.[7]

Now 98 centers are functioning under the Safe Blood Transfusion program in Bangladesh. All centers are provided with the basic equipment and furniture which is necessary for running a blood transfusion service. The development of manpower, supply of reagent and mandatory screening of TTI (five infections) are also included under this program. There are two types of Transfusion Centers in Bangladesh. One is the public blood centers operating at different hospitals. They are controlled by the government regulations and supported fully by the Ministry of Health and Family Welfare (MOH) and FW. They are operating according to the bylaw formulated by the MOH and FW. The other type of blood centers are private blood centers. Previously there was a specific rule for them but at present they come under the same bylaw formulated by the MOH and FW and they have to obtain a license from the DGHS (Director General of Health Services) office to operate.

It is mandatory to perform five TTI tests on donated units. All private and public centers are supposed to perform all five mandatory TTI tests and quality control system is supposed to be maintained by Safe Blood Transfusion program. User’s fee (service changes) for one bag of blood in a public blood bank varies from US$ 3 to US$7 according to the economic status of the patient whereas in private blood banks it varies from US$ 6 to US$ 12. The funds generated by this method are used for the development of the transfusion services in the country. It is also observed that there is an increased trend for use of blood components like concentrated Red Cell, FFP, and Platelet concentrate etc. in the country.

There is an acute shortage of trained manpower in the transfusion centers. So, steps have been taken to increase the number of staff and simultaneously training is going on by Safe Blood Transfusion Program (SBTP) regularly to upgrade the knowledge and skill of the manpower. It is observed that transmission of TTI is also because of biomedical waste disposal . Proper disposal of biomedical waste is being taken care of in three areas like general waste, infected waste (sharp), infected waste (non-sharp).

New strategies have been adopted for the development of blood transfusion services in Bangladesh. There is phase-wise upgradation of the centers across the country. There is a plan for strengthening six Blood Transfusion Centers to state-of-the-art centres (L1) through provision of facilities for 100% voluntary blood donation and blood component preparation. To strengthen 13 other Blood Transfusion Centers at standard of (L2), is underway and an agreement has already been signed between the MOH and FW and WHO through the assistance of World Bank and Department For International Development (DFID) to upgrade the 13 centers. Now phase-wise up-gradation of the centers is going on. There is target-based program and expansion of the activities in Bangladesh BTS. The situation in BTS has been improving continuously for the last few years. To provide the best quality of service, every center should follow the rules and regulations of the new gazette and the recommendations of international agencies strictly.

Now the Bangladesh BTS is receiving help from WHO, the International Society of Blood transfusion (ISBT) and the South Asian Association of Transfusion Medicine (SAATM) to achieve targets. It is widely expected that Bangladesh BTS will be able to supply safe blood from voluntary blood donors to all parts of the country in timely manner in sufficient quantity.
